# Structural features of PhoX, one of the phosphate-binding proteins from Pho regulon of *Xanthomonas citri*

**DOI:** 10.1371/journal.pone.0178162

**Published:** 2017-05-22

**Authors:** Vanessa R. Pegos, Rodrigo M. L. Santos, Francisco J. Medrano, Andrea Balan

**Affiliations:** 1 Universidade Estadual de Campinas – UNICAMP, Instituto de Biologia (IB), Campinas, São Paulo, Brazil; 2 Centro Nacional de Energia e Materiais (CNPEM), Laboratório Nacional de Biociências (LNBio), Campinas, São Paulo, Brazil; 3 Universidade de Mogi das Cruzes – UMC, Mogi das Cruzes, São Paulo, Brazil; 4 Universidade Federal de São Paulo – UNIFESP, Diadema, São Paulo, Brazil; 5 Centro de Investigaciones Biológicas (CSIC), Madrid, Spain; 6 Universidade de São Paulo (USP), Instituto de Ciências Biomédicas II (ICBII), São Paulo, São Paulo, Brazil; Russian Academy of Medical Sciences, RUSSIAN FEDERATION

## Abstract

In *Escherichia coli*, the ATP-Binding Cassette transporter for phosphate is encoded by the *pstSCAB* operon. PstS is the periplasmic component responsible for affinity and specificity of the system and has also been related to a regulatory role and chemotaxis during depletion of phosphate. *Xanthomonas citri* has two phosphate-binding proteins: PstS and PhoX, which are differentially expressed under phosphate limitation. In this work, we focused on PhoX characterization and comparison with PstS. The PhoX three-dimensional structure was solved in a closed conformation with a phosphate engulfed in the binding site pocket between two domains. Comparison between PhoX and PstS revealed that they originated from gene duplication, but despite their similarities they show significant differences in the region that interacts with the permeases.

## Introduction

Phosphorus is an essential nutrient for all living organisms as a component of nucleic acids, plasma membranes, enzymes, and in other elementary functions of the cell. Due to its biological relevance, bacteria have developed specialized systems for phosphorus uptake such as the low-affinity transporter, PitA, and the Phosphate Specific Transporter (Pst), an ATP-Binding Cassette transporter (ABC transporter). The Pst system belongs to the well-characterized Pho regulon, which comprises at least 32 genes involved in adaptive response under phosphate starvation [1, 2, 3], which has been correlated with oxidative stress, cell surface modification, activation of virulence, environmental adaptation, adhesion, and pathogenesis [4].

Structurally, the Pst system consists of two transmembrane proteins, two associated cytoplasmic ATPases and a periplasmic protein responsible for the affinity and specificity of the system. In *Escherichia coli*, the periplasmic binding protein is PstS, an alpha/beta protein consisting of two domains connected by a hinge region that allows for the movement of ligand binding. In ABC transporters, besides the uptake and transport of substrates, periplasmic components have an important role in signaling and induction of conformational changes that occur in transmembrane and cytoplasmic domains for transport [5].

Previously, we characterized the putative Pho regulon proteins from the plant pathogenic bacterium *Xanthomonas citri*, the causative agent of citrus canker disease, and showed the presence of two putative phosphate-binding proteins, PstS (XAC1577) and PhoX (XAC1578) that share 70% amino acid sequence identity [6]. Proteomic studies revealed that under phosphate limitation, PhoX and PstS were highly induced with fold change values of 43.5 and 39.1 respectively, indicating that both proteins have an active role in the Pst system. Furthermore, we showed that *pstS* and *phoX* genes are under the control of different promoters supporting the differences in the expression levels and regulation of the proteins. However, despite all investigation, the specific function of each protein is still not clear. In addition to phosphate uptake, PstS participates in the gene regulation of *E*. *coli* [1], *Pseudomonas sp*. [7, 8], *Proteus mirabilis* [9], *Synechocystis sp*. [10], biofilm formation and chemotaxis in *P*. *aeruginosa* [11, 12]. In all of these bacteria, there is no additional phosphate-binding protein, which makes *X*. *citri* PhoX an interesting case.

To characterize PhoX in comparison with PstS, we built a phylogenetic tree including orthologues and paralogues from distinct classes, and carried out structural analyses using different approaches. Our results showed that PstS and PhoX originated from gene duplication and horizontal transfer from a common ancestor, and that many other microorganisms have more than one putative phosphate-binding protein. The three-dimensional structure of PhoX was solved in the P2_1_ space group, in a closed conformation (bound to phosphate), with eight molecules present in the asymmetric unit. Comparing the structure of PhoX to the model of PstS showed conservation of all residues involved in the phosphate interaction, but revealed differences in the electrostatic potential of the putative regions that interact with the membrane proteins. Furthermore, the proteins showed slight structural changes after phosphate addition and significant differences in the thermal resistance. The structural characterization of both proteins will elucidate new aspects in phosphate transport and regulation in *X*. *citri*.

## Materials and methods

### Plasmid construction, protein expression and purification

Amplification and cloning of the *phoX* gene was performed according to Pegos and collaborators [13]. The *pstS* gene encoding the mature PstS protein, without the first N-terminal 20 amino acids, was amplified by PCR using the following primers (Forward: 5'- CATATGGCGTGCTCGCCCGGCAAG -3' and Reverse 5'-CTCGAGTTACTTGAACTCGCTGCCCC -3'), which contain *Nde*I and *Xho*I restriction enzyme sites, respectively (underlined). The PCR reaction was carried out in a final volume of 50 μL, following the HiFi *Taq* DNA polymerase protocol (Invitrogen) and analyzed using 0.8% agarose gel electrophoresis. The fragment of 1032 nucleotides was cloned into pGEM T-Easy vector (Promega) and subcloned into pET28a(+) expression vector (Novagen) to build the pET28a_pstS plasmid. The cloning was confirmed by DNA sequencing and the plasmid was used for transformation of *E*. *coli* Tuner (DE3) competent cells. Protocols for PstS expression and purification were performed as previously described for PhoX by Pegos and collaborators [13] and the results are shown in S1 Fig (Supporting information). Both proteins were concentrated to 30 mg/mL using centrifugal 10 MWCO filters (Amicon Millipore).

### Spectroscopy analysis

Circular dichroism measurements of PhoX and PstS were carried out in a Jasco J-810 spectropolarimeter (Jasco) equipped with a Peltier type temperature controller. Spectra were obtained in 1 mm path length quartz cell using a protein concentration of 2 μM (0.13 mg/mL and 0.09 mg/mL for PhoX and PstS, respectively) in 10 mM Tris-HCl buffer at pH 8.0 in the absence and the presence of 30 μM NaH_2_PO_4_. The far-UV CD spectra were collected from 260 nm to 190 nm with 35 consecutive scans and the average spectra were corrected by subtracting the buffer and ligand contribution. The observed ellipticity was converted into mean residue ellipticity [θ] based on a mean residue molecular mass of 98.2 for PstS and 105.1 for PhoX. The secondary structure was estimated using the Dichroweb package [14]. In addition, the thermal unfolding was followed at 222 nm by increasing the temperature from 20°C to 95°C and the midpoint of the curve was considered the unfolding transition. Protein concentration was calculated according to Edelhoch [15].

### Determination of the three-dimensional structure of the PhoX and molecular modeling of PstS

Crystals of PhoX that diffracted up to 3.0 Å resolution were obtained in 20% PEG 3350 and 200 mM sodium iodine and belonged to the P2_1_ space group [13]. Data were processed using the XDS package [16], merged and scaled with Aimless [17] and Free-R flag column was added using Uniqueify (CCP4i). The Matthews coefficient was 2.25 Å^3^/Dalton, which corresponded to a 45% of solvent content, most likely indicating the presence of 8 monomers of PhoX in the asymmetric unit [13]. The structural coordinates of the *E*. *coli* phosphate-binding protein PstS [18] (PDB code 2ABH), which shares 60% of amino acid sequence identity with *X*. *citri* PhoX, was prepared with Chainsaw [19] and used as a search model. Molecular replacement was performed with Phaser [20] and Buccaneer [21] was used for the automated model building. Twinned refinement (domain 1: twin operator H, K, L and twin fraction 0.628 and domain 2: twin operator -H, -K, L and twin fraction 0.372) was carried out using REFMAC [22] to a final Rfree/Rfactor of 0.18/0.24. The atomic coordinates have been deposited in the PDB under 5I84 accession code. The statistics of refinement are shown in S1 Table.

To compare the structures of PhoX and PstS, a model of PstS was built based on the structural coordinates of *X*. *citri* PhoX (PDB 5I84), which shares 70% amino acid sequence identity with PstS. Twenty models were built using Modeller [23] and the best one was chosen based on the free energy and quality of the stereochemical parameters. The structural superposition of the proteins was performed in COOT [24] using the secondary structure-matching tool [25]. Figures and analyses of proteins were prepared using Pymol (The Pymol Molecular Graphisc System, version 1.5.0.4, Schröndinger, LLC).

### Phylogenetic and evolutionary relationships of *X*. *citri* PstS and PhoX

To analyze the phylogenetic relationship of *X*. *citri* PstS and PhoX, a list of different microorganisms was chosen based on the presence of PstS, PhoX or both proteins from alpha, beta, gamma and delta-proteobacteria groups. As the outgroup member, we chose *Thermanaerovibrio acidaminovorans*, a representative of the Synergistetes group. The accession numbers and species are described in S2 Table. First, we searched for microorganisms and orthologues using the String server [26] and then performed a psi-BlastP of *X*. *citri* PstS and PhoX amino acid sequences against the genome of the reference organism. The choices were confirmed and sequences were included when they showed at least 20% amino acid sequence identity over a minimum of 50% query coverage, and the gene annotation or function related to phosphate binding. Amino acid sequences were previously analyzed using the SEAL software [27] aligned by Muscle [28] and then manually refined based on the secondary structure. Phylogenetic reconstructions were built with the MEGA 6 software [29] using distance and statistical reconstructions methods. For the statistical method, we used a LG+G model of amino acid substitution indicated by Mr. Modeltest [30] as most appropriate. Support indices were obtained by 5000 bootstrap replicates.

## Results

### The PstSBAC ABC transporter and two-component system PhoR-PhoB are highly conserved in proteobacteria and PhoX and PstS originated from a gene duplication in *X*. *citri*

*X*. *citri* PhoX and PstS amino acid sequences were used to search orthologues among the members of the proteobacteria branch using Blastp. The presence and conservation of PhoX and PstS orthologues was identified in distinct members of the proteobacteria clade, including microorganisms that live in a variety of habitats such as gastrointestinal and respiratory tracts, human mucosa, ocean and marine sediments, water, citrus, soil, and roots (Table 1). Members of the α-, β-, and ε-proteobacteria groups, with exception of *Thiobacillus denitrificans*, presented one phosphate-binding protein with two orthologues that shared around 50% amino acid sequence identity with *X*. *citri* PstS and PhoX. In δ-proteobacteria, only *Desulfatibacterium alkenivorans* has two orthologues (dal_Dalk1427_PBP and dal_Dalk2845_PBP), which shared 24% and 23% identity with *X*. *citri* PstS and PhoX, respectively. Finally, in γ-proteobacteria, the presence of two or more orthologues of the phosphate-binding protein is common. In this clade, the proteins shared an amino acid sequence identity with PstS and PhoX ranging from 24% to 93%. As expected, higher values were obtained among orthologues from the closely related species *X*. *citri*, *X*. *campestris* and *S*. *maltophilia*, which also revealed the presence of two proteins dedicated to phosphate binding. Besides the presence of up to 4 phosphate-binding proteins, all of the microorganisms analyzed presented the components of Pst system dedicated to phosphate uptake and the orthologues of the two-component system PhoR-PhoB (S2 Table).

**Table 1:** Occurrence of phosphate-binding proteins in proteobacteria and their comparison with PstS and PhoX from *X*. *citri*. *X*. *citri* PhoX was used as entry for String server and the data of cooccurrence were analysed for building of the table. The presence of orthologs of PhoX and PstS was avaluated in the four branches of proteobacteria branch. To obtain the amino acid sequence identity among the proteins, the sequences of the proteins identified by String server were submitted to BLASTp x *X*. *citri* database. The protein identification shows the 3-letter code of the microrganism, KEGG number and the function associated to the putative protein (PBP: phosphate-binding protein; PstS: phosphate-specific transporter).

	Identified proteins and amino acid sequence identity with PstS			Identified proteins and amino acid sequence identity with PhoX			
Microorganism	KEGG code and protein name	Amino acid Sequence Identity	E-value	KEGG code and protein_name	Amino acid Sequence Identity	E-value	Habitat or Environment
α**-proteobacteria**							
*Paracoccus denitrificans*	no homology with PstS	-	-	pde_PDEN4330_PBP	26%	0,05	Soil
*Sphingopyxis alaskensis*	sal_SALA0826_PBP	25%	4,00E-06	no homology with PhoX	-	-	Marine sediments
*Sphingomonas wittichii*	swi_Swit_1104_PBP	53%	1,00E-99	swi_Swit_1104_PBP	55%	5,00E-117	Water, root and soil
β**-proteobacteria**							
*Thiobacillus denitrificans*	tbd_TBD1136_PBP tbd_TBD1420_PBP	48% 50%	3,00E-94 1E-97	tbd_TBD1136_PBP tbd_TBD1420_PBP	50% 49%	1,00E-96 7E-96	Soil and Marine sediments
*Nitrosospira multiformis*	nmu_NMULA0486_PBP	20%	4,00E-07	no homology with PhoX	-	-	Soil
*Nitrosomonas europaea*	neu_NE0531_PBP	25%	1,00E-06	no homology with PhoX	-	-	Soil
γ**-proteobacteria**							
*Xanthomonas axonopodis* pv. *citri*	xac_XAC1577_PstS	100%	0,00E+00	xac_XAC1578_PhoX	100%	0,00E+00	Soil and citrus
*Escherichia coli*	eco_b3728_PstS	49%	3,00E-99	eco_b3728_PstS	55%	7,00E-117	Gastrointestinal
*Yersinia pestis*	ype_YPO4117_PstS ype_YPO3203_PstS	50% 23%	3,00E-101 0.68	ype_YPO4117_PstS ype_YPO3203_PstS	56% -	2,00E-118 -	Gastrointestinal
*Pseudomonas aeruginosa PAO1*	pae_PA5369_PstS	24%	6,00E-04	no homology with PhoX	-	-	Soil and citrus
*Xanthomonas campestris 33913*	xcc_XCC1527_PstS	94%	0,00E+00	xcc_XCC1528_PhoX	93%	0,00E+00	Soil and citrus
*Shewanella oneidensis MR1*	son_SO4292_PstS	23%	1,00E-4	son_SO4292_PstS	25%	7,00E-07	Ocean
*Haemophilus influenzae*	hin_HI1383m_PstS	57%	1,00E-130	hin_HI1383m_PstS	55%	2,00E-119	Respiratory tract
*Xylella fastidisiosa*	xfa_XF2141_PBP	77%	0,00E+00	xfa_XF2141_PBP	68%	9,00E-152	Citrus
*Stenotrophomonas maltophilia*	sml_Smlt1552_PBP	80%	0,00E+00	sml_Smlt1554_PstS	80%	8,00E-118	Human mucose
*Vibrio cholerae N16961*	vch_VCA0070_PBP	28%	6,00E-05	vch_VCA0070_PBP	24%	8,00E-05	Diverse ecosystems
*Photobacterium profundium*	no homology with PstS ppr_PBPRA1394_PBP	- 28%	- 2,00E-04	ppr_PBPRB0883_PBP no homology with PhoX	26% -	4,00E-05 -	Marine sediments
*Kangiella Koreensis*	no homology with PstS	-	-	kko_KKO2087_PBP	24%	7,00E-05	Ocean
*Nitrosococcus oceani*	noc_NOC0584_PBP	25%	8,00E-05	noc_NOC0584_PBP	28%	2,00E-09	Ocean
δ**-proteobacteria**							
*Anaeromyxobacter dehalogenans*	ade_ADEH4006_PBP	29%	4,00E-06	ade_ADEH4006_PBP	26%	9,00E-04	Soil and marine sediments
*Sorangium cellulosum*	scl_SCE2946_PstS	48%	2,00E-85	scl_SCE2946_PstS	45%	3,00E-82	Soil and animal faeces
*Desulfatibacterium alkenivorans*	dal_Dalk1427_PBP	24%	2,00E-06	dal_Dalk2845_PBP	23%	3,00E-06	Marine sediments
*Syntrophus aciditrophicus*	sat_SYN02226_PBP	27%	1,00E-07	sat_SYN02226_PBP	26%	0,001	sewage treatment plant
*Lawsonia intracellularis*	lip_LI1028_PBP	32%	2,00E-15	lip_LI1028_PBP	29%	3,00E-09	Animal small intestine
ε**-proteobacteria**							
*Sulfurospirillum deleyianum*	sde_SDEL1859_PBP	27%	1,00E-03	sde_SDEL1859_PBP	26%	3,60E+00	Water
*Nautilia profundicola*	Nam_NAMH0308	40%	1,00E-66	Nam_NAMH0308	41%	7,00–75	Deep Sea
*Sulfurovum sp*.	sun_SUN2272_PstS	41%	2,00E-06	sun_SUN2272_PstS	42%	4,00E-72	Marine sediments
**Synergistetes**							
*Thermanaerovibrio acidaminovorans*	tai_Taci0095_PBP	27%	8,00E-02	tai_Taci0095_PBP	24%	6,00E+00	Sugar refinary reactor

**Fig 1:**
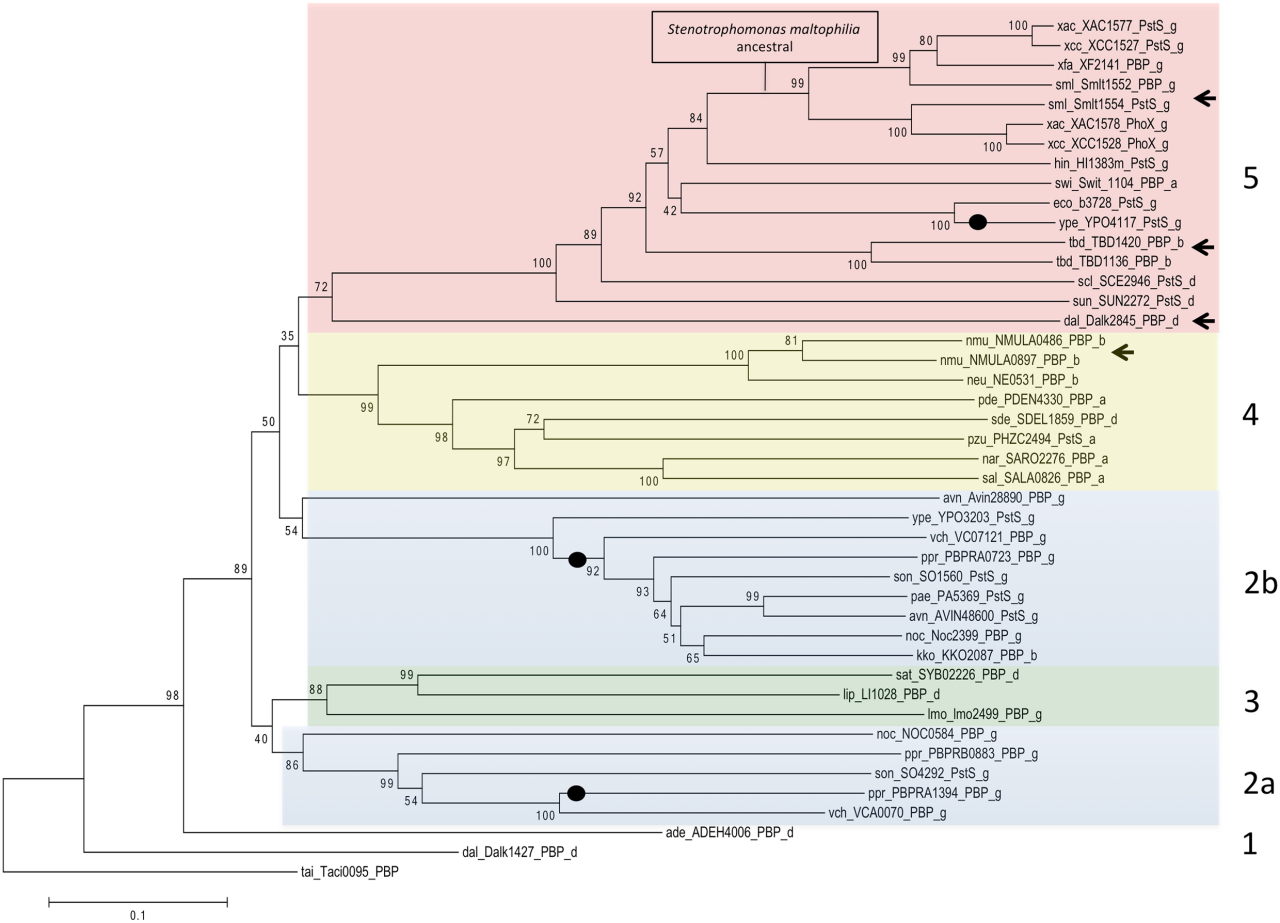
Phylogenetic relationships between the orthologues of PstS and PhoX. Five groups classify the distinct orthologues found in proteobacteria. All the organisms used in this tree as well as their reference codes are described in Table 1. The tree was generated using MEGA 6.0 software and the neighbor-joining algorithm according MM section. The numbers at the nodes indicate the bootstrap percentages of 1000 replicates. Arrows and black dots indicate gene duplication and horizontal gene transfer, respectively. The colors highlight the different groups and subgroups.

The neighbor-joining tree obtained for those taxa (Fig 1) showed the phylogenetic relationship and origin of PstS and PhoX proteins. The ancestral gene of the phosphate-binding proteins is similar to the orthologue from *D*. *alkenivorans*, a δ-proteobacteria that presented two copies of PstS: Dalk1427 and Dalk2845. Dalk1427 seems to be related to PstS from the five groups, including the orthologue from the γ-proteobacteria *Anaeromyxobacter dehalogenans*, AdeH4006 (group 1).

The four other monophyletic groups showed uncertain relationships between each other: groups 2a and 2b (Fig 1, blue) include both orthologues found in *Nitrosococcus oceani*, *Photobacterium profundium*, *Shewanella oneidensis* MR1 and *Vibrio cholerae* N16961, whereas 2b should had been acquired by horizontal transfer from *Yersinia pestis*; group 3 (Fig 1, green) includes *Lawsonia intracellularis* and *Syntrophus aciditrophicus*; group 4 (Fig 1, yellow) with orthologues of α-, β- and δ-proteobacteria is strongly supported by the bootstrap values, and group 5 (Fig 1, red), that includes the *D*. *alkenivorans* (Dalk2845) paralogue, originated from a gene duplication event (shown with arrows). Duplications were also evident in group 4, represented by *Nitrosospira multiformis* proteins, and group 5. Within group 5, the duplication event of the genes encoding the phosphate-binding protein of *S*. *maltophilia* (Fig 1, box) originated in the PstS and PhoX lineages. PstS of *X*. *citri* belongs to PBP clade of *S*. *maltophilia* and PhoX to PstS clade.

The phylogeny also suggests at least three events of horizontal gene transfer (Fig 1, black dots): (i) between *E*. *coli* and *Yersinia pestis* (references b3728 and YPO4117, respectively); (ii) between *V*. *cholerae* and *P*. *profundium*, which is the only taxa that showed three phosphate-binding protein genes, and (iii) between *Y*. *pestis* (ype_YPO3203_PstS) and Group 2a. Due to the small bootstrap values between the groups, it is not possible to discount a shared duplication between 2a and 2b and the loss of a paralogous copy (2a) in *Y*. *pestis*. Nonetheless, the conflicting relationship between paralogues of group 2a compared to group 2b does not corroborate this hypothesis.

### The three-dimensional structure of *X*. *citri* PhoX was solved in a closed conformation bound to phosphate

The structure of PhoX was solved in the P2_1_ space group with 8 molecules in the asymmetric unit (S2A Fig, Supporting information). The organization of the molecules in the crystal suggests some oligomerization from the monomers that formed dimers (chains A-G, C-E, C-D, E-F, D-F) and then tetramers (chains C-D-E-F) (S2A Fig, Supporting information). Analysis of the protein interfaces with PISA (PDBePISA, http://www.ebi.ac.uk/msd-srv/prot_int/cgi-bin/piserver) has not revealed any specific interactions that could result in the formation of stable quaternary structures. Still, the analysis suggests that the structures likely do not form a complex in solution and the oligomerization observed is probably due the crystal packing.

**Fig 2:**
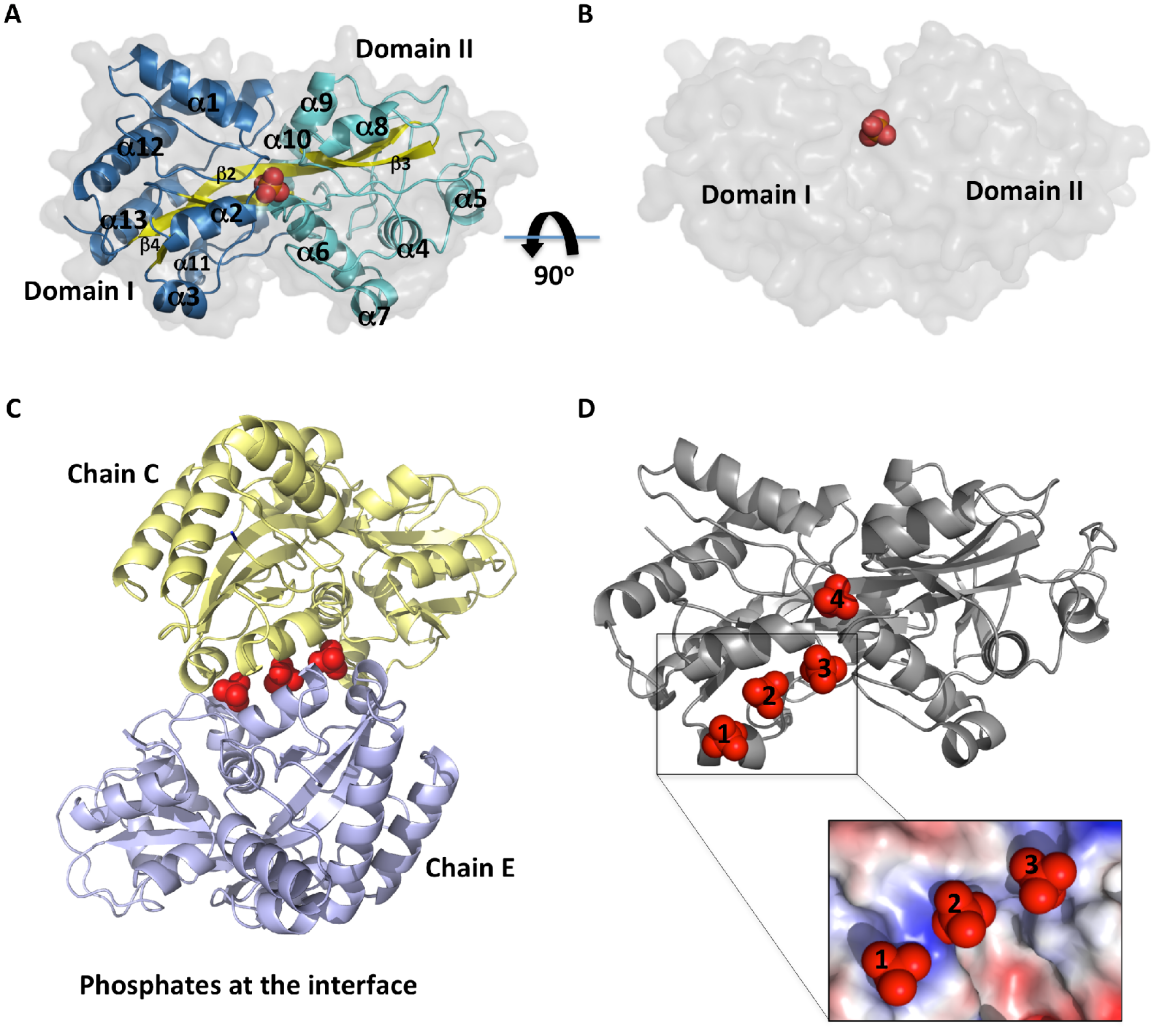
Crystal structure of the phosphate-binding protein PhoX from *X*. *citri*. (A) Cartoon representation of the three-dimensional structure of PhoX evidencing the α/β folding and the surface (transparent gray) α-helices and loops from domains I and II are shown in blue and cyan, respectively. The β-sheet is colored in yellow. All the secondary structures are labeled. Phosphate ions are shown as red spheres. (B) Side-view of PhoX in surface with the ion buried inside the ligand-binding pocket between both domains. (C) Protein-protein interactions between chains C and E as observed in the crystallographic structure of PhoX, evidencing the set of three phosphates (red spheres) mediating the interaction. (D) Positioning of phosphates 1, 2 and 3 in domain I of chain E, and phosphate 4 inside the ligand-binding pocket. In the box: details of the positive electrostatic potential.

The structure of PhoX is similar to the phosphate-binding proteins with two domains (I and II, Fig 2A, blue and cyan, respectively), each one consisting of a three strand β-sheet (Fig 2A, yellow) surrounded by 7 and 5 α-helices, respectively in domains I and II. Domain I is formed by three segments consisting of residues 26 to 100, 252 to 265 and 277 to 340, and domain II is formed by the continuous residues 101 to 251.

Six out of the eight molecules in the asymmetric unit were in the same closed conformation with a phosphate ion inside the ligand-binding pocket located between both domains (Fig 2B). Although there was no electron density for any ligand in two of the molecules, structurally they also showed the typical closed conformation of the phosphate bound protein. The hinge region between both domains is made up by a 16 residue long β-strand (β2, residues 93 to 108) that, most likely, limits the PhoX flexibility in comparison to other phosphate-binding proteins. That characteristic included PhoX in the type II substrate-binding proteins, according to Berntsson and collaborators [31]. Besides the phosphate inside the pocket, a set of inline phosphates positioned at domain I was evident in all the protein molecules in the asymmetric unit (S2B Fig, Supporting information). These phosphates mediated crystal contacts between the molecules through hydrogen bonds with residues in α-helix 2 at domain I (Fig 2C), which shows a positive electrostatic potential (Fig 2D).

### The ligand-binding pocket in *X*. *citri* PhoX is highly conserved

**Fig 3:**
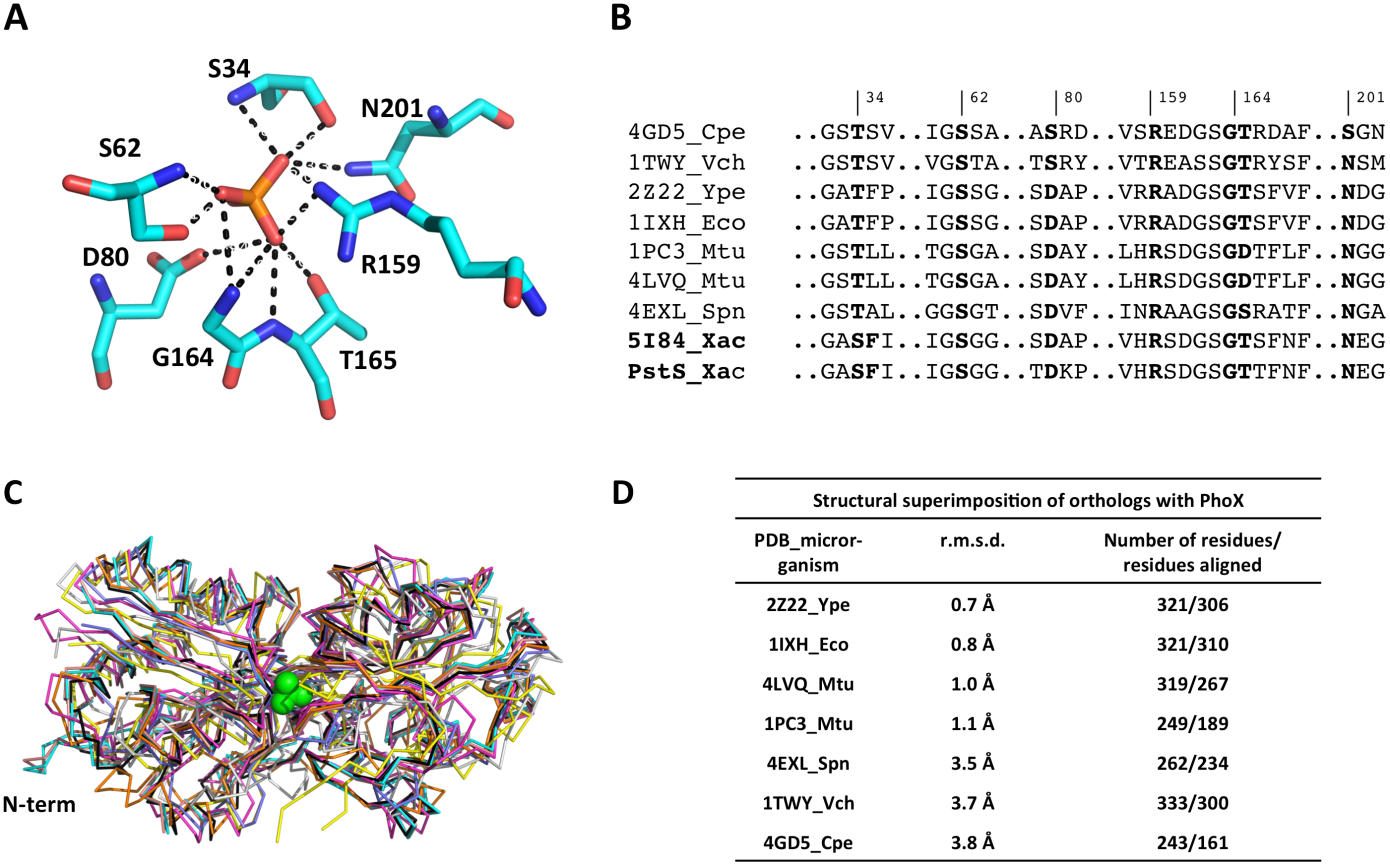
The ligand-binding pocket of *X*. *citri* PhoX and its conservation. (A) Detailed view of the PhoX residues (cyan sticks) that interact with the phosphate ion (red stick). Hydrogen bonds are shown as black trace. (B) Structural based amino acid sequence alignment of *X*. *citri* PhoX and PstS with several orthologues with solved structures, showing the high level of conservation of the residues that interact with the ion. Numbers are shown according to PhoX structure. (C) Structural superimposition of *X*. *citri* PhoX (in black ribbon) and all of the phosphate-binding proteins structures as deposited in PDB. Proteins are shown as ribbons and the phosphate ion from *X*. *citri* structure as green spheres. (D) R.m.s.d. values and the aligned residues after the structural superposition of PhoX (314 residues) and each protein is shown in angstroms. PDB codes and 3-letters represent the following proteins: 4GD5_Cpe, *Clostridium perfringens* PBP, (light gray); 1TWY_Vch, *Vibrio cholerae* PBP (light blue); 2Z22_Ype, *Yersinia pestis* PstS (cyan); 1IXH_Eco, *E*. *coli* PstS (yellow); 1PC3_Mtu, *M*. *tuberculosis* PstS1 (blue); 4LVQ_Mtu, *M*. *tuberculosis* PstS3 (magenta), 4EXL_Spn, *Streptococcus pneumoniae* PstS1 (green), 5I84_Xac, *X*. *citri* PhoX (black) and PstS_Xac, *X*. *citri* PstS (red).

The phosphate inside the ligand-binding pocket of *X*. *citri* PhoX is coordinated through 12 hydrogen bonds performed by seven residues: Ser34, Ser62, Asp80, Arg159, Gly164, Thr165 and Asn201 (Fig 3A) highly conserved in the orthologues with solved structures (Fig 3B).

Only in *X*. *citri* proteins, a threonine is replaced by serine in position 34. Furthermore, the position of Asp80 in *X*. *citri* PhoX is sequentially and structurally conserved when compared to *E*. *coli* PstS and *Mycobacterium tuberculosis* PstS3. In the *E*. *coli* orthologue, this aspartate is responsible for the recognition of the phosphate ion and its discrimination among other tetrahedral oxyanions [32, 33]. In the *Mycobacterium tuberculosis* PstS3, this residue plays an important role in mono- and dibasic phosphate binding and discrimination between phosphate and sulfate species [34]. Confirming the results from the phylogenetic analysis that showed *Y*. *pestis* and *E*. *coli* as the closest orthologues of PhoX, the superimposition of these structures revealed the lowest values for the r.m.s.d. Accordingly, the proteins of *Clostridium perfringens*, *Vibrio cholerae* and *Streptococcus pneumoniae* presented the higher values of r.m.s.d. (Fig 3C and 3D).

### PstS and PhoX from *X*. *citri* show differences in the regions that interact with the membrane components

**Fig 4:**
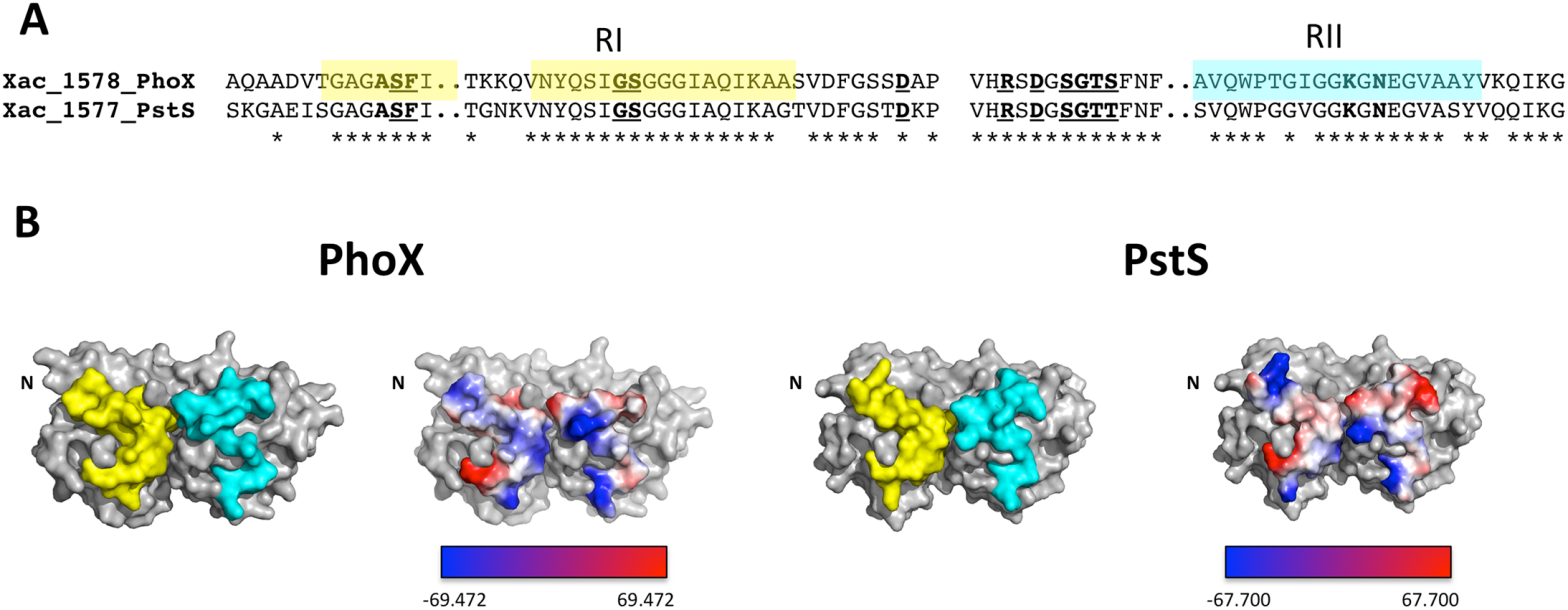
Comparison and analysis of the regions of PhoX and PstS that interact with the permeases PstCA. (A) Amino acid sequence alignment of RI and RII from PhoX, PstS and *E*. *coli* PstS evidencing the high level of conservation (asterisks). Residues in underlined bold are involved with the phosphate binding. (B) The localization of the RI (yellow) and RII (cyan) regions of PhoX and PstS close to the entrance of the ligand pocket shown in surface view and electrostatic potential. Proteins are shown in the same orientation. Electrostatic potential of RI and RII of PhoX and PstS are shown in colored view surface according to the charges calculated in Pymol. Negative: red, positive: blue and neutral: white.

A comparison between PhoX and PstS proteins from *X*. *citri* was performed to gather information regarding their structural differences and similarities. In a previous paper, we showed that the *phoX* gene did not belong to the operon that included *pstSCABphoU* genes encoding the ATP-Binding Cassette transporter for phosphate uptake and the negative regulator of the system, the protein PhoU. In addition, we showed that PstS and PhoX were upregulated, at different levels, under phosphate starvation [6]. Since all residues involved in ligand binding were conserved in the two proteins (Fig 3A), we looked to determine if the interface of these proteins that interact with the membrane components PstC and PstA were similar. The differences in the electrostatic potential in the regions that interact with the permeases might affect the protein-protein interactions, which are dependent on many factors including residues, surface availability, and charges. Two regions, denominated RI and RII for domains I and II, respectively, were defined in PhoX and PstS and analyzed according to their amino acid sequence similarity and electrostatic potential (Fig 4).

The sequence alignment showed that RI and RII (Fig 4A, in yellow and cyan, respectively) present only some residues not conserved in PhoX and PstS. In this comparison, three out of twenty-six residues from RI and four out of twenty residues from RII were different (Fig 4A). In RI, the changes are conservative (Ala for Gly and Ser for Thr) but in RII, they are quite different (Ala for Ser and Thr for Gly). These differences in the sequence changed the electrostatic potential of RI and RII of both proteins (Fig 4B), suggesting they might have different affinities for the permeases PstC and PstA.

### Phosphate interaction in PhoX and PstS of *X*. *citri* induces an increase in thermal stability

**Fig 5:**
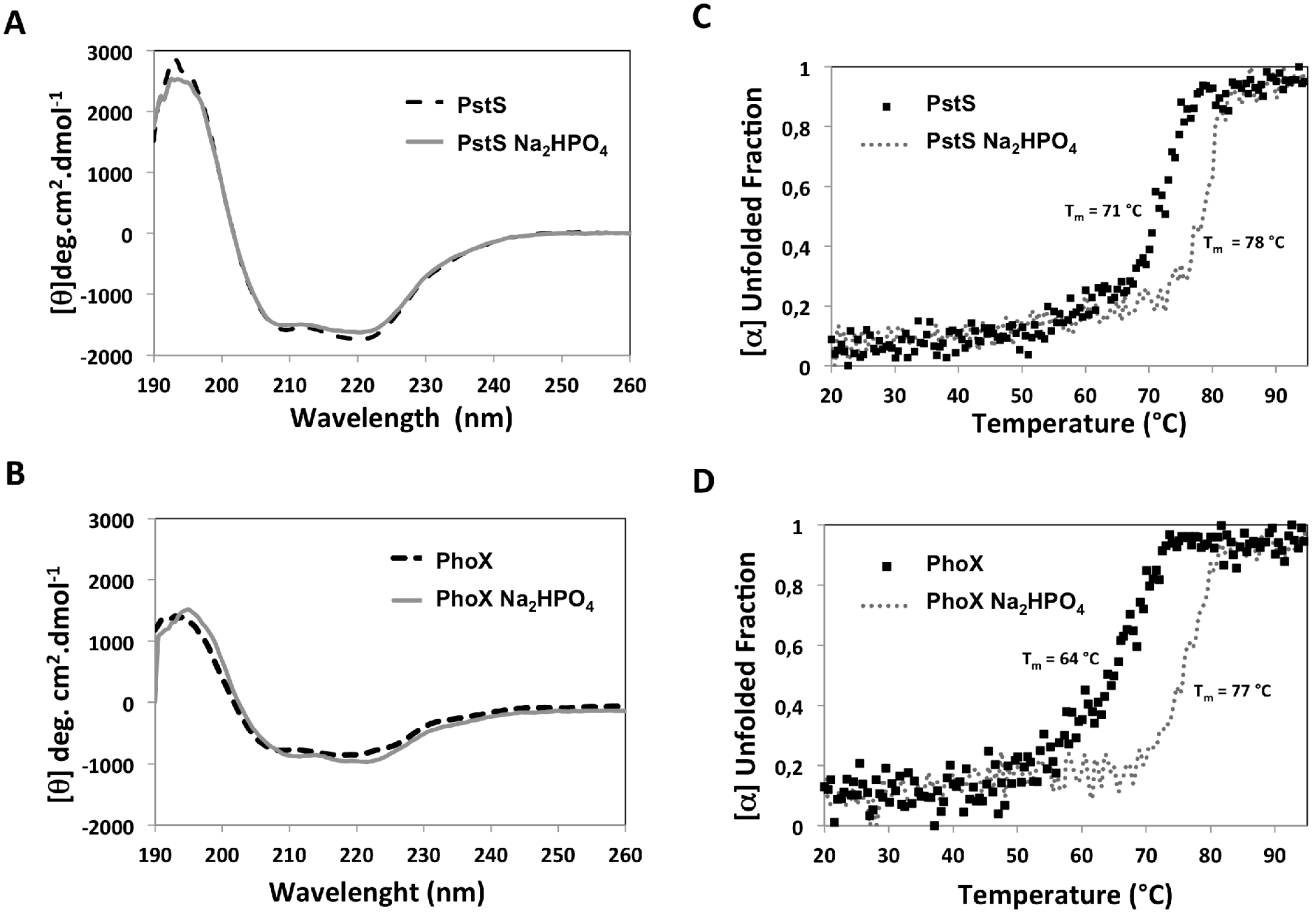
Spectroscopic analysis of *X*. *citri* PstS and PhoX in presence and absence of phosphate. Secondary structure prediction of PstS (A) and PhoX (B) was measured by circular dichroism. Traced line: PstS and PhoX in the absence of phosphate; Black line: PstS and PhoX in the absence of phosphate. Gray line: PhoX and PstS incubated with 30 μM of phosphate. The thermal stability of the proteins in absence (black dots) and presence (gray dots) of phosphate was measured at 222 nm for PstS (C) and PhoX (D). The melting temperature (Tm) was calculated and is shown for both conditions.

The secondary structure and folding of PhoX and PstS in the absence and the presence of phosphate was compared using circular dichroism (CD). The far-UV CD spectra of both proteins, without and with phosphate, are similar, consisting of a maximum at 195 nm and two relative minima at 208 and 222 nm, which corroborates the expected α/β fold of PhoX and model of PstS (Fig 5A and 5B).

The prediction of the secondary structure content, carried out with the program Selcon3 on the Dichroweb server showed that the helical content of PstS is higher than that of the PhoX while this protein has higher β-sheet content (Table 2). A comparison of the orthologues with the solved three-dimensional structure also showed those differences in the secondary structure content among the proteins.

**Table 2:** Comparison of the secondary structure content of PhoX (crystallographic structure) and PstS (molecular model) from *X*. *citri* with ortologues that have solved three-dimensional structure. Secondary structure prediction was performed by Selcon3-SP175 (190 nm -240 nm) at Dicroweb.

Protein Name	Microorganism	Number of Residues	Amino acid sequence identity (%) related to PhoX	PDB code	Secondary structure content (%)			Reference
					α-helix	β-δηεετ	Loops	
**PhoX**^a^	***Xanthomonas citri***	315	100	**5I84**	**19/41**	**30/14**	**44/45**	This work
PstS	*Xanthomonas citri*	343	70	-	22	28	50	This work
PstS	*E*.*coli*	341	51	1IXH	38	22	40	[32]
PstS	*Yersinia pestis*	321	52	2Z22	39	23	38	[39]
PstS1	*Mycobacterium tuberculosis*	350	33	1PC3	34	18	48	[40]
PstS	*Vibrio cholerae*	290	28	1TWY	30	23	47	To be published

Secondary structure prediction was performed by Selcon3-SP175 (190 nm -240 nm) at Dicroweb.

^a^ Percentage of CD analysis and protein structure (CD/structure).

The most significant change was observed upon thermal denaturation of the proteins. The presence of phosphate increased the melting temperature of the proteins (64°C to 77°C for PhoX, and 71°C to 78°C for PstS) (Fig 5C and 5D). In accordance with the three-dimensional structure, PhoX is bound to phosphate by 12 hydrogen bonds, which would lead to the increasing in the thermal stability (Fig 3A).

## Discussion

Phosphate is an elementary macronutrient for bacterial growth not freely abundant. To overcome the depletion of phosphate, prokaryotic species developed specialized systems for the uptake of phosphate, such as the Pit system, where the transport of phosphate is associated to a divalent metal cation and the proton-motive force [35]; and the largely studied Pst system, an ATP-dependent transporter (ABC transporter) of high affinity. Despite the knowledge of these transporters in bacteria, lack of information is given to this system in the *Xanthomonas* genus. In this work, we focused on the characterization of the *X*. *citri* phosphate-binding protein PhoX and comparison with its paralogue PstS.

To perform the genome analyses and search of genes, we first observed that there is no consensus for the nomenclature PstS, PhoX or PBP and that the three terms correspond to phosphate-binding proteins without a clear definition. Based on phylogenetic analysis of orthologues of PhoX and PstS that belong to different species of Proteobacteria, we identified that both proteins originated from a gene duplication that occurred in an ancestral orthologue from *D*. *alkenivorans* and then again, in the *Xanthomonas* sp. group, since two copies of these genes were found in *X*. *citri* and *X*. *campestris*. Gene acquisitions in bacteria that share a similar environment might also originate from distinct mechanisms of horizontal gene transfer such as transduction, transformation and conjugation. Many other paralogous sequences might have been continually created, replaced or lost during the evolution of this system and this seems to be the case in the absence of support for a close relationship between Gamma 1 and Gamma 2 Groups. Indeed, we observed that the presence of the periplasmic component(s) and its cognate ABC transporter is followed in all the samples by the presence of the two-component system formed by PhoR and PhoB proteins and PhoU. This fact is in accordance with the evidence found in Firmicutes that shows a clear coevolutionary relationship between ABC permeases and histidine kinases from two-component systems [36]. Moreover, Moreno-Letelier and collaborators [37] also showed parallel evolution and horizontal gene transfer of the *pst* operon in Firmicutes living in oligotrophic environments.

Up to four copies of genes encoding phosphate-binding proteins were also evidenced in the genome of *Protobacterium sp*., *Thiobacillus denitrificans* and *Yersinia pestis*, bacteria that inhabit very competitive environments and with low phosphate availability, and changes in the ionic force and pH [38]. The presence of multiple periplasmic-binding proteins largely increases the chances of phosphate uptake and the velocity of the transport giving to the cells higher competitive skills. In all the species studied in this work, it is noteworthy that the active site is highly conserved as observed in PstS and PhoX, corroborating their putative functions, but similarly, the paralogues diverge in other regions of the sequences such as RI and RII suggesting differences in the mechanisms of interaction, uptake and transport. RI and RII in PstS and PhoX are quite similar in both proteins when sequences are aligned, but comparing the charge profile resultant from the structure and model, we observe that they show differences in the electrostatic potential that will probably induce a different behavior with the permeases in the membrane. Important questions arise from that analysis: (i) what about the affinity of PstS and PhoX?; (ii) how does the affinity affect the velocity of the transport?; (iii) could PhoX and PstS could induce different structural conformations?; (iv) if PstS is also associated with regulation, what happens when a bacterium has more than one phosphate-binding protein? In *P*. *aeruginosa*, PstS has a dual function in transport and chemotaxis and interacts, besides with the permeases from the cognate transporter, with the CtpL protein, a membrane receptor [12]. Searching for the orthologues of CtpL in *X*. *citri* identified a set of proteins that share low sequence identity but that are putatively involved in chemotaxis. Could PstS and PhoX be capable of interacting with other receptors in *X*. *citri*? Certainly, evidence shown in this work is intriguing and stimulates further analysis of the role of PstS and PhoX in *X*. *citri*.

Regarding the three-dimensional structure of PhoX compared to the orthologues, no significant differences can be discerned, despite the presence of a longer β-strand joining both domains and sustaining the basis of the ligand-binding site. This secondary structure might be responsible for the higher thermal stability when compared to PstS, evidenced in the CD assays in the presence of phosphate (5°C), and also for the very little differences observed between the bound and unbound conformational states presented by the chains in the asymmetric unit. Similar results using far UV CD spectroscopy were obtained with PstS from *P*. *aeruginosa* in the bound and unbound states [12], indicating that the proteins do not suffer significant structural changes after ion binding.

The crystallographic structure of PhoX revealed the presence of additional phosphates, facilitating interactions between the chains. None of the phosphate-binding proteins with available structure in the PDB presented similar organization of the ions, probably as a result of the crystallographic packing, which is not evident in other orthologues since they are presented as a monomer or dimer.

## Conclusions

Finally, this work shows the first structure of PhoX, an ABC transporter component involved with phosphate uptake from a phytopathogenic bacterium, which could be used as a model in the *Xanthomonas* genus, since the orthologues from different species share high amino acid sequence identity. We also compared PhoX with its paralogue, PstS, and showed that they are highly conserved in the ligand-binding pocket but show significant differences in their thermal stability after ion interaction, and in their surface electrostatic potential. These data suggest the transport and mechanisms of protein interaction might be different for both proteins.

## Supporting information

S1 Fig Purification of *X*. *citri* phosphate-binding protein PstS. (A) Immobilized metal affinity chromatography of PstS using Nickel column. Lane 1: molecular marker; lane 2: Flowthrough; lane 3: wash step with Tris-Cl 20 mM pH 8.0; Imidazole; lane 4: elution fraction with 50 mM imidazole; lane 5: elution fraction with 100 mM imidazole; lane 6: 500 mM imidazole. (B) Size-exclusion chromatography in column Hi Load 16/60 200 superdex (GE Healthcare Life Science). The elution was performed using 10 mM of Tris-Cl pH 8.0. Two peaks were obtained representing the aggregates and monomeric states, respectively, of the proteins. The inset gel is showing the Comassie staining gel of the purified samples in each peak. Peak 2 was used for further experiments.
(PDF)

S2 Fig Crystal packing of *Xanthomonas citri* PhoX in the asymmetric unit. (A) The structural organization of the eight molecules in the asymmetric unit. (B) Positioning of the phosphates in the different chains from the crystallographic structure of *X*. *citri* PhoX. Phosphates were evidenced always in the RI region from domain I mediating crystal contacts between the chains.
(PDF)

S1 Table Refinement statistics of the *Xanthomonas citri* PhoX structure. (PDF)

S2 Table Occurrence of proteins belonging to the putative phosphate ABC transporter (Pst system) and the two-component system (PhoR-PhoB) in proteobacteria. The protein identification was based on the sequence homology after BlastP of the *X*. *citri* proteins against the sequence data bank. The functional protein association networks String was used to support the presence, identity and relationship among the proteins.
(PDF)
