# Supplementary figures and images for: Structural features of PhoX, one of the phosphate-binding proteins from Pho regulon of Xanthomonas citri
Source: PLoS One. 2017 May 22;12(5):e0178162. doi: 10.1371/journal.pone.0178162 (PMC5439949; doi:10.1371/journal.pone.0178162)

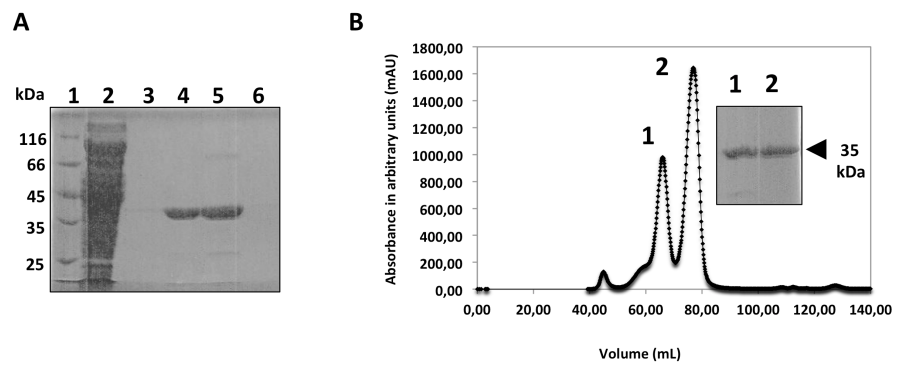

**S1 Fig**

Supplement: S1 Fig — (A) Immobilized metal affinity chromatography of PstS using Nickel column. Lane 1: molecular marker; lane 2: Flowthrough; lane 3: wash step with Tris-Cl 20 mM pH 8.0; Imidazole; lane 4: elution fraction with 50 mM imidazole; lane 5: elution fraction with 100 mM imidazole; lane 6: 500 mM imidazole. (B) Size-exclusion chromatography in column Hi Load 16/60 200 superdex (GE Healthcare Life Science). The elution was performed using 10 mM of Tris-Cl pH 8.0. Two peaks were obtained representing the aggregates and monomeric states, respectively, of the proteins. The inset gel is showing the Comassie staining gel of the purified samples in each peak. Peak 2 was used for further experiments. (PDF) [file pone.0178162.s001.pdf]

**A**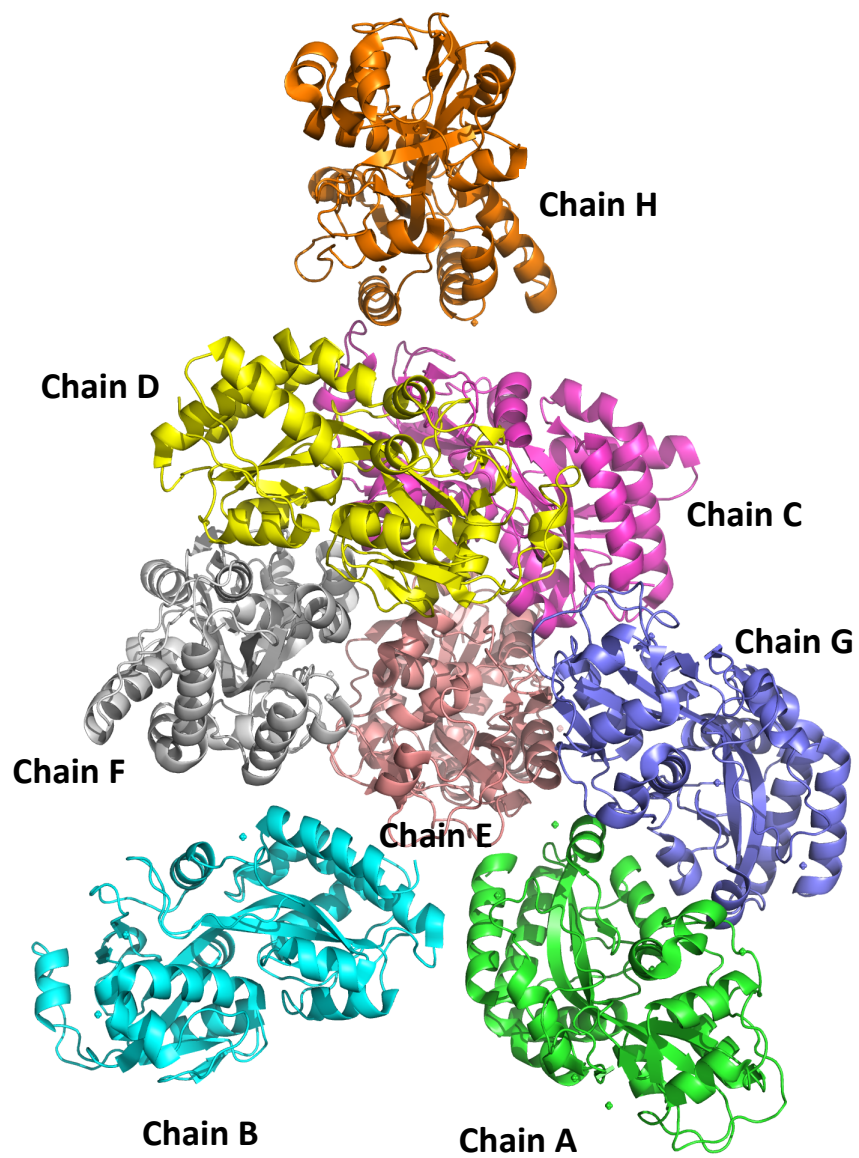**B**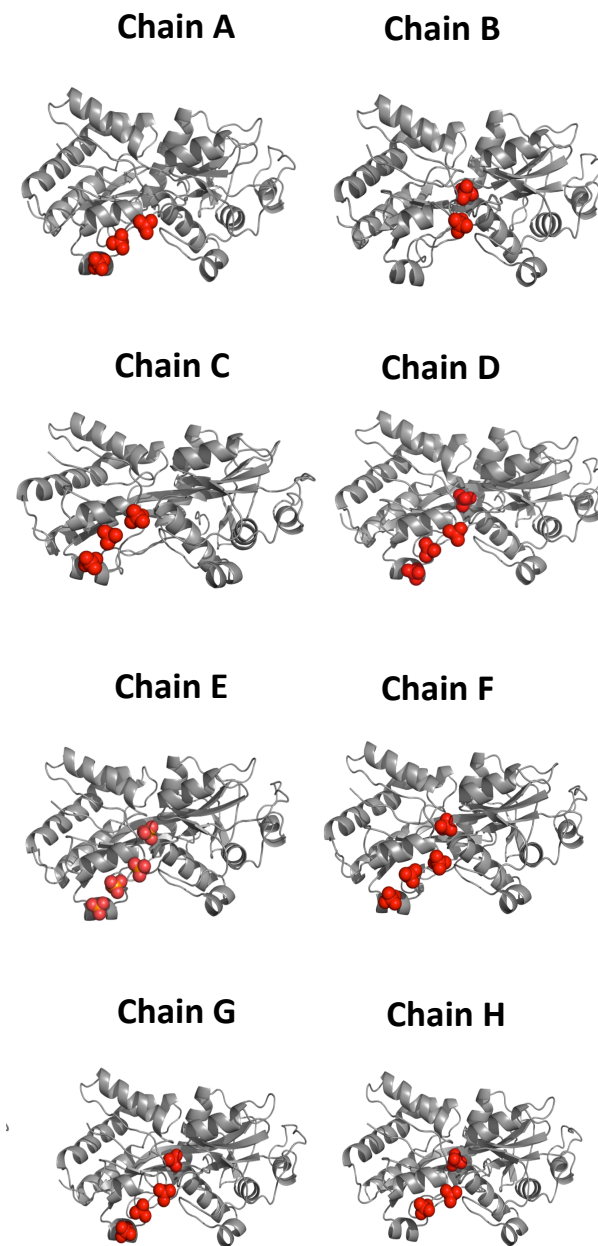**S2 Fig.**

Supplement: S2 Fig — (A) The structural organization of the eight molecules in the asymmetric unit. (B) Positioning of the phosphates in the different chains from the crystallographic structure of X. citri PhoX. Phosphates were evidenced always in the RI region from domain I mediating crystal contacts between the chains. (PDF) [file pone.0178162.s002.pdf]
