# Supplementary material for: Structural features of PhoX, one of the phosphate-binding proteins from Pho regulon of Xanthomonas citri
Source: PLoS One. 2017 May 22;12(5):e0178162. doi: 10.1371/journal.pone.0178162 (PMC5439949; doi:10.1371/journal.pone.0178162)
Supplement: S1 Table — (PDF) [file pone.0178162.s003.pdf]

S1 Table

Data Refinement	
Resolution range high (Å)	2.98
Resolution range low (Å)	17.06
Completeness for range (%)	92.2
Number of reflections	44.230
R-factor	0.178
R-free	0.249
Average B-factor ($Å^2$)	24.83
water	28
Protein residues	314
RMSD (bonds) (Å)	0.019
RMSD (angles) (Å)	2.17
Ramachandran favored	2275
Ramachandran allowed	205
Ramachandran outliers	8
