# Supplementary material for: Structural features of PhoX, one of the phosphate-binding proteins from Pho regulon of Xanthomonas citri
Source: PLoS One. 2017 May 22;12(5):e0178162. doi: 10.1371/journal.pone.0178162 (PMC5439949; doi:10.1371/journal.pone.0178162)
Supplement: S2 Table — The protein identification was based on the sequence homology after BlastP of the X. citri proteins against the sequence data bank. The functional protein association networks String was used to support the presence, identity and relationship among the proteins. (PDF) [file pone.0178162.s004.pdf]

S2 Table

		Two component System		ABC Transporter					Regulator	Porin
Organism	Org code	PhoB	PhoR	PstS	PhoX or PstS2	PstA	PstC	PstB	PhoU	OprO
**α-proteobacteria**										
Paracoccus denitrificans	pde	PDEN4325	PDEN3938	PDEN4330	-	PDEN4328	PDEN4329	PDEN4327	PDEN4326	-
Novosphingobium aromaticivorans	nar	SARO2282	SARO2275	SARO2276	-	SARO2277	SARO2278	SARO2279	SARO2280	-
Sphingopyxis alaskensis	saI	SALA0821	SALA0822	SALA0826	-	SALA0824	SALA0825	SALA0823	SALA0822	-
Sphingomonas wittichii	swi	SWIT3577	SWIT1099<br>SWIT3575	SWIT1104	SWIT1104	SWIT1102	SWIT1103	SWIT1101	SWIT3576	-
Phenylobacterium zucineum	pzu	PHZ507	PHZ0501<br>PHZ0502	PHZ2494	-	PHZ0504	PHZ0503	PHZ0503	PHZ505	PHZ1100<br>PHZ3312
**β-proteobacteria**										
Thiobacillus denitrificans	tbd	TBD0216	TBD0217	TBD1420<br>TBD1136	TBD1420<br>TBD1136	TDB1138	TDB1137	TDB1136	TBD0025	-

Nitrosospira multiformis	nmu	NMUIA2231	NMUIA2232	NMUIA0486	NMUIA0897	NMUIA1086	NMUIA1087	NMUIA1088	NMULA0435	NMULA089<br>8
Nitrosomonas europaea	neu	NE2131	NE1288	NE0531	-	NE0999	NE1000	NE1001	NE1744	-
					**γ-proteobacteria**					
Xanthomonas axonopodis pv. *citri*	xac	XAC1042	XAC1041	XAC1577	XAC1578	XAC1575	XAC1576	XAC1574	XAC1573	XAC3484
Escherichia coli K-12 MG1655	eco	B0399	B0400	B3728	B3728	B3726	B3727	B3725	B3724	-
Yersinia pestis	ype	YPO3205	YPO3204	YPO3203<br>YPO4117	YPO3203<br>YPO4117	YPO4115	YPO4116	YPO4114	YPO4113	-
Pseudomonas aeruginosa PAO1	pae	PA5360	PA5361	PA5369 PA0688	-	PA5367	PA5368	PA5366	PA5365	-
Xanthomonas campestris 33913	xcc	XCC0963	XCC0962	XCC1527	XCC1528	XCC1525	XCC1526	XCC1524	XCC1523	XCC1529
Azotobacter vinelandii	avn	AVIN_48520	AVIN_48530	AVIN_48600<br>AVIN_28890	-	AVIN_48580	AVIN_48590	AVIN_48570	AVIN_48560	-
Shewanella oneidensis MR1	son	SO_1558	SO_1559	SO_4292<br>SO_1560	-	SO_4291	SO_4290	SO_4289	-	SO_1557
Haemophilus influenzae	hin	HI1379	HI1380	HI1383	-	HI1381	HI1382	HI1380	-	-
Xylella fastidisiosa	xfa	XF2593	XF2592	XF2141	-	XF2142	XF2143	XF2144	XF2145	-

Stenotrophomonas maltophilia	sml	SMLT0978	SMLT0977	SMLT1552	SMLT1554	SMLT1550	SMLT1551	SMLT1549	SMLT1548	SMLT1555
Vibrio Cholerae N16961	vch	VC0719	VC0720	VC07121<br>VC0070	-	VC0724	VC0725	VC0726	VC0727	-
Photobacterium profundium	ppr	PBPRA0721	PBPRA0722	PBPRA0723<br>PBPRA0775<br>PBPRA 0883<br>PBPRA1394	-	PBPRA0728<br>PBPRA1392	PBPRA0729<br>PBPRA1393	PBPRA1391<br>PBPRA0730	PBPRA0731	-
Kangiella Koreensis	kko	KKOR2094	KKO2093	-	KKO2087	KKOR2098	KKO2097	KKOR2096	KKOR2095	KKOR2099
Nitrosococcus oceani	noc	NOC2400	NOC2401	NOC2399<br>NOC0584	NOC0584	NOC0580<br>NOC2398	NOC0582<br>NOC2397	NOC0581<br>NOC2396	NOC2395	-
**δ-proteobacteria**										
Anaeromyxobacter dehalogenans	ade	ADEH4009	ADEH4008	ADEH4006	-	ADEH4005	ADEH4004	ADEH4003	ADEH4002	-
Sorangium cellulosum	scl	SCE2948<br>SCE5073	SCE2947<br>SCE3423<br>SCE7574	SCE2946	-	SCE2944	SCE2945	SCE2943	SCE2942	-
Desulfatibacillum alkenivorans	dal	DALK1425	DALK1426	DALK1427<br>DALK2845	-	DALK1428	DALK1429	DALK1430	DALK1431	-
Sulfurospirillum deleyianum	sdl	-	-	SDEL1859	-	ADEl1858	SDEL1857	SDEL1856	268680491	-

Sulfurovum sp.	sun	SUN2268	SUN2267	SUN2272	SUN2272	SUN2270	SUN2271	SUN2269	SUN2273	-
**Mollicutes**										
Mycoplasma genitalium	mge	-	-	MG412	-	MG411	-	MG410	MG409	-
**Synergistetes**										
Thermanaerovibrio acidaminovorans	tai	TACI0090	TACI0089	TACI0095	-	TACI0093	TACI0094	TACI0092	TACI0091	-
